# Single or Double Plating for Acromial Type III Fractures: Biomechanical Comparison of Load to Failure and Fragment Motion

**DOI:** 10.3390/jcm11113130

**Published:** 2022-05-31

**Authors:** Marianne Hollensteiner, Sabrina Sandriesser, Felix Rittenschober, Josef Hochreiter, Peter Augat, Lukas Ernstbrunner, Reinhold Ortmaier

**Affiliations:** 1 Institute for Biomechanics, BG Unfallklinik Murnau gGmbH, 82418 Murnau, Germany; marianne.hollensteiner@bgu-murnau.de (M.H.); sabrina.sandriesser@bgu-murnau.de (S.S.); biomechanik@bgu-murnau.de (P.A.); 2 Institute for Biomechanics, Paracelsus Medical University, 5020 Salzburg, Austria; 3 Department of Orthopedic Surgery, Ordensklinikum Linz Barmherzige Schwestern, Vinzenzgruppe Center of Orthopedic Excellence, Teaching Hospital of the Paracelsus Medical University Salzburg, 4020 Linz, Austria; felix.rittenschober@ordensklinikum.at (F.R.); josef.hochreiter@ordensklinikum.at (J.H.); 4 Department of Orthopaedic Surgery, Royal Melbourne Hospital, Parkville, VIC 3052, Australia; lukas.ernstbrunner@gmx.at; 5 Melbourne Orthopaedic Group, Windsor, VIC 3181, Australia; 6 Department of Biomedical Engineering, University of Melbourne, Parkville, VIC 3010, Australia

**Keywords:** reverse total shoulder arthroplasty, scapula, acromial fracture, osteosynthesis, biomechanical, failure load, interfragmentary motion

## Abstract

Background: Acromial Levy III fractures after inverse shoulder arthroplasty occur in up to 7% of patients. To date, it is not clear how these fractures should be treated as clinical outcomes remain unsatisfactory. The aim of this study was to evaluate the biomechanical performance of three different plating methods of type III acromion fractures. Methods: Levy III fractures in synthetic scapulae were fixed with three different methods. Angular stable locking plates were placed on the spina scapula to bridge the fracture either dorsally, caudally, or on both aspects by double plating. In a biomechanical experiment, the pull of the deltoid muscle at 40° abduction of the arm was simulated by cyclic loading with increasing load levels until failure. Failure load, cycles to failure, and fragment motions were evaluated. Results: The results showed that double plating (350 ± 63 N) withstood the highest loads until failure, followed by dorsal (292 ± 20 N) and caudal (217 ± 49 N) plating. Similarly, double plating showed significantly smaller fragment movement than the other two groups. Conclusions: Double plating appeared to provide the largest biomechanical stability in type III acromion fracture under arm abduction. Caudal plating in contract resulted in insufficient fracture stability and early failure and can thus not be recommended from a biomechanical point of view.

## 1. Introduction

Acromial fractures are relatively common following reverse shoulder arthroplasty and are reported to occur in 1 to 7% of patients [1,2,3]. A cause leading to fracture is increased traction of the deltoid muscle due to the changed position of the arm in relation to the scapula. Osteoporotic bone structure, onlay-humeral- and lateralized-glenoid-prosthesis designs and predetermined fractures caused by fixation screws in the metalback are described to be risk factors for acromial fractures after reverse shoulder arthroplasty [4,5,6,7].

**Figure 1:**
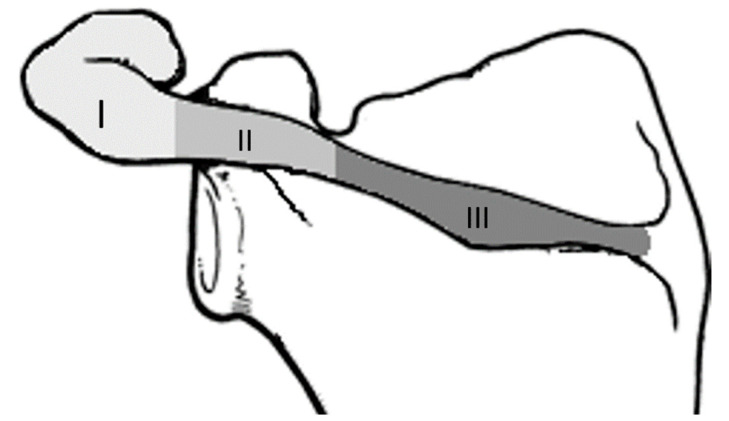
Illustration of the Levy classification of acromial fractures.

According to the classification of Levy et al., acromial fractures can be divided into three types depending on the location of the fracture: acromial tip fractures (type I), fractures posterior to the ac joint (type II), and acromial base fractures (type III) [8] (see Figure 1). Acromion fractures can occur after falls or as stress fractures [9,10]. Type I and II fractures can be treated conservatively with acceptable results [2,3,11]. To stabilize a type III fracture properly is a challenge due to the bony shape of the scapular spine and the broad deltoid muscle insertion [8,12,13]. To date, it is not clear how type III fractures should be treated. Clinical outcomes after acromial fractures in combination with reverse shoulder arthroplasty are heterogeneous and mostly poor [2,14]. Nonoperative treatment often results in nonunion or malunion with poor functional outcomes [14]. On the other hand, surgical treatment does not necessarily show better results than conservative therapy [13].

Clear surgical treatment recommendations are not available. High failure rates due to nonunion and implant failure have been described for surgical procedures. However, literature is scarce and often it is not clear which fracture types were treated and which surgical techniques were used [13,14]. One popular treatment option is to place a singular plate from the cranial side. Alternatively, the plate can be applied from the dorsal aspect or from both sides in a double plating approach [15].

The purpose of this study was to determine and evaluate the biomechanical performance of three different plating methods of acromial type III fractures with special regard to failure loads, failure modes, and interfragmentary motion. We hypothesized that for acromial type III fractures, double plate osteosynthesis is biomechanically superior to single-plate fixation.

## 2. Materials and Methods

### 2.1. Specimen Preparation

In order to determine the mechanical performance of three different plating methods of acromial fractures, eighteen synthetic scapulae (scapula, large, left, 4th generation, #3413, Sawbones, Malmö, Sweden) were used as a substrate. Type III acromial fractures, according the classification of Levy et al. [8], involving the middle and deltoid origin of the acromion, were created with an oscillating saw. In order to perform the fracture identically in all synthetic scapulae, a cutting template was fabricated. According to the saw blade thickness, a 0.8 mm fracture gap remained.

**Figure 2:**
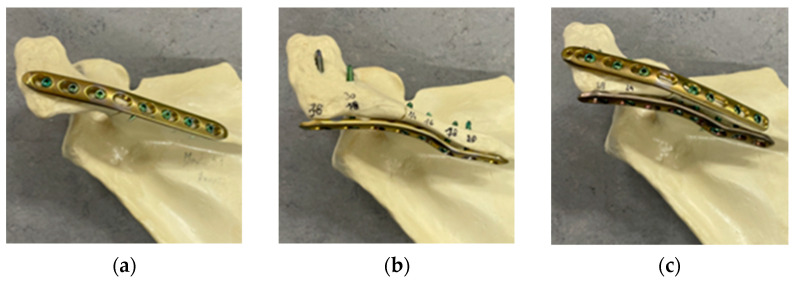
Three plating methods were investigated: (**a**) LCP placed below spina scapulae (LCPcaudal), (**b**) LCP placed on backside of spina scapulae (LCPdorsal), and (**c**) a double plating approach (LCPdouble).

The fractured scapulae were randomly assigned to three groups (n = 6) and were treated with different osteosynthesis constructs. The first group (LCPcaudal) was treated with an 8-hole locking compression plate (LCP, 111 mm, 423.581, DePuy Synthes, Warsaw, IN, USA) and six self-tapping locking screws (diameter 3.5 mm, DePuy Synthes, Warsaw, IN, USA). The plate was positioned below the spina scapulae. The screws were placed in a clinically relevant manner with varying lengths (see Figure 2a). The two most laterally located screws extended cranially into the acromion, and four medially located screws were screwed into the spina of the scapula. The most medial hole of the LCP remained free. The same LCP was used in the second group (LCPdorsal) and was placed dorsally on the spine of the scapula. Three screws were placed in the lateral part of the plate, extending anteriorly in the acromion, and four screws were placed in the spine of the scapula (see Figure 2b). The third group (LCPdouble) corresponds to a combination of the first two groups (see Figure 2c) using a double plating technique.

To minimize unwanted variation in fracture care or plate positioning and to ensure comparability and reproducibility in all experiments, custom-made templates were used for plate precontoring and plate placement on the scapula.

### 2.2. Biomechanical Testing

To ensure reproducible, physiological force transmission in the dynamic electrical testing machine (Instron E3000, Instron, Norwood, MA, USA), the specimens were clamped in an anatomically correct position with a negative mold made of polyurethane resin (RenCast FC52/53, Huntsman Advanced Materials, Basel, Switzerland). Due to the fact that the anatomical position of the scapula is dependent on arm movements, the scapula was positioned in the test machine with 15° of internal rotation, 13° of upward rotation, and 0° of anterior tilt to mimic the natural position of the scapula during 40° of arm abduction [16].

**Figure 3:**
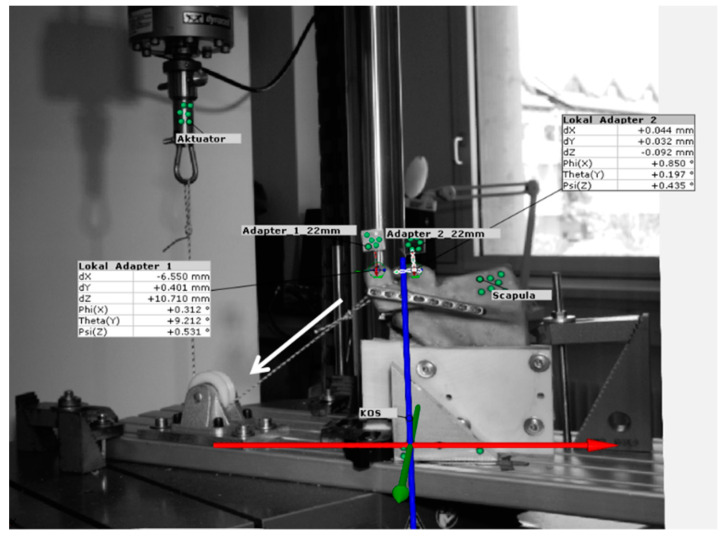
Test setup and superimposed 3D motion analysis. Stiffness and fragment movements of the acromial fragment were evaluated in the direction of the muscle pull of the deltoid muscle (white arrow). The co-ordinate system (blue, red, and green arrows) reflects the anatomical spatial planes.

An inelastic dyneema cord (SG0075, 2 mm area, Best Divers srl, Rezzato, Italy), guided over a pulley and attached to the force sensor of the testing machine, simulated the pull of the deltoid muscle during arm abduction (Figure 3). The cord was looped around the acromion so that the direction of traction corresponds to the resultant pulling direction of the pars acromialis of the deltoid muscle [17]. Before testing, marker dots were placed on the specimens to track the motion of the acromial fragment in relation to the fixed scapula with a 3D camera system (Aramis, GOM, Braunschweig, Germany).

A pulling load protocol was adapted from Kicinski et al. [15], simulating the physiological loading on the acromion applied through the traction of the pars acromialis of the deltoid muscle [18].

In order to determine the construct stiffness prior to dynamic testing, the test specimens were quasistatically loaded five times with load ramps to 50 N at 0.01 mm/s [15]. Stiffness was evaluated in the direction of the muscle’s pull. Therefore, the slopes between 30 and 70% of the maximum load from the linear region of the load-displacement curves were averaged from the last three ramps, while the first two ramps were used for settling of the setup.

Then, cyclic loading was applied with a valley load of 25 N and a peak load of 50 N for 1000 cycles. The peak load was increased by 25 N every 1000 cycles and was applied at a frequency of 2 Hz. The load was increased until failure, which was defined as bone, screw, or implant breakage. Load and cycles to failure were recorded and failure mechanisms were photo-documented. During cyclic loading, the movements of the acromial fragment in relation to the fixed scapula were evaluated at each load increment.

### 2.3. Statistical Analysis

For statistical analyses, box plots were created and mean and standard deviation were also computed for each group. For comparisons between plating methods, ANOVA and Tukey post hoc corrections were computed with SPSS (SPSS Statistics, Version 26, IBM, Armonk, NY, USA).

## 3. Results

### 3.1. Stiffness of Constructs

**Figure 4:**
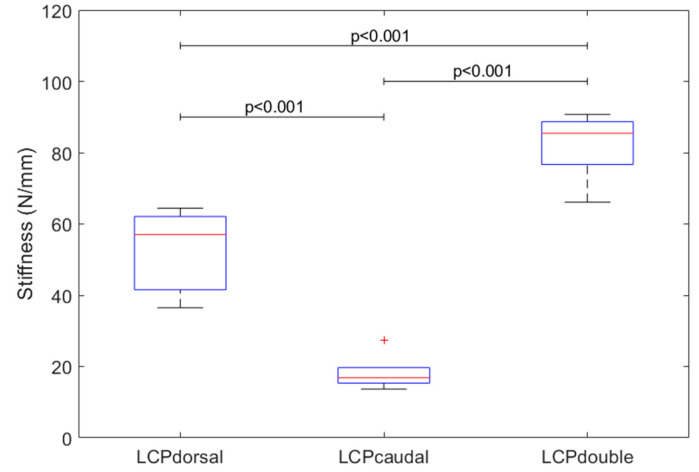
Stiffness of the three LCP constructs (boxplot shows median (red line), interquartile range (box), and maxima and minima (upper and lower whisker)).

Construct stiffness differed among the three constructs (*p* < 0.001). LCPcaudal was found to be the most flexible (18 ± 5 N/mm), followed by LCPdorsal (53 ± 11 N/mm) and LCPdouble (82 ± 9 N/mm). Thus, the stiffness of LCPdorsal was almost three times that of LCPcaudal (*p* < 0.001). The stiffness of LCPdouble was around 55% greater than that of LCPdorsal (*p* < 0.001) and 355% higher than LCPcaudal (*p* < 0.001, Figure 4).

### 3.2. Failure Load and Cycles

**Figure 5:**
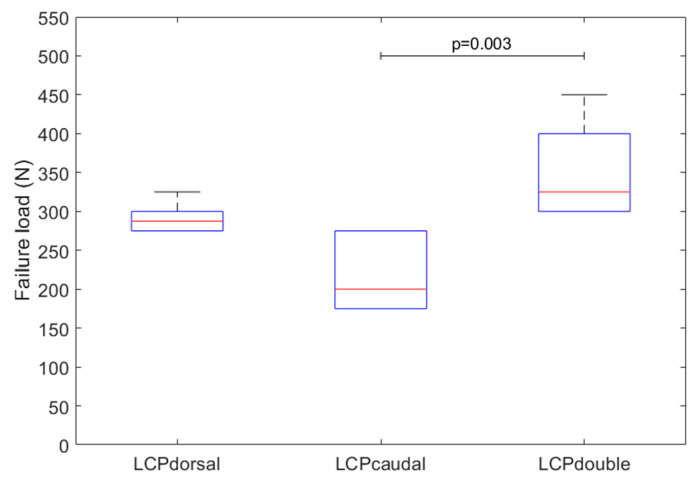
Loads to failure of the three investigated constructs (boxplot shows median (red line), interquartile range (box), and maxima and minima (upper and lower whisker)).

Failure load (*p* = 0.004, Figure 5) and cycles to failure (*p* = 0.003) differed among the three constructs. LCPcaudal failed at the lowest loads (217 ± 49 N, 7243 ± 2063 cycles), followed by LCPdorsal (292 ± 20 N, 10249 ± 1040 cycles). LCPdouble with two plates withstood the highest loads and thus the most load cycles (350 ± 63 N, 12559 ± 2457 cycles). The failure load for LCPdorsal was around 35% greater than for LCPcaudal (*p* = 0.13). The failure loads of LCPdouble were around 62% higher than those of LCPcaudal (*p* = 0.003) and around 20% higher than those of LCPdorsal (*p* = 0.16).

### 3.3. Motion of Acromial Fragment

**Figure 6:**
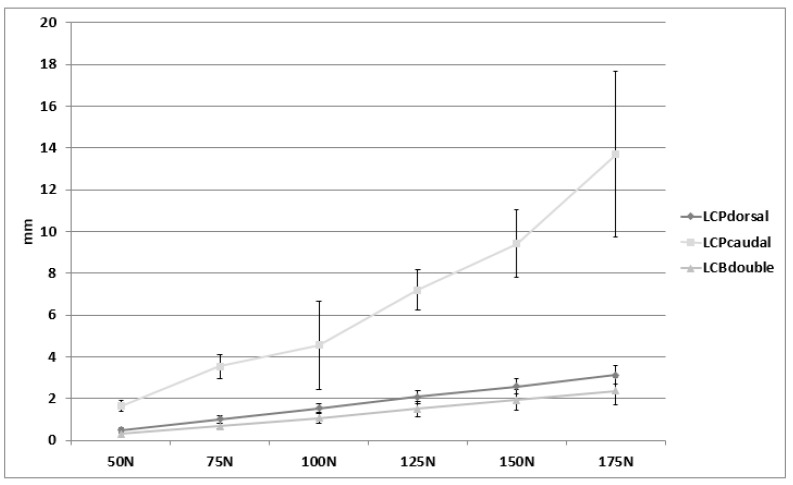
Interfragmentary motion (mean and standard deviation) of the acromion over several load levels (50 to 175 N) of the three constructs.

According to the publication by Ackland et al., the deltoid muscle pulls with up to 175 N during an arm abduction of 40° (based on a person weighing 75 kg) [19]. Therefore, the movements of the acromion fragment were evaluated at each increment up to a load level of 175 N and in the direction of the deltoid’s muscle pulling direction (Figure 3). The course of the movements over all load levels is shown in Figure 6.

Fragment motion differed among the three constructs (*p* < 0.001). LCPdouble showed the smallest movement of the acromial fragment, closely followed by the LCPdorsal (*p* = 0.7). The two groups showed a flat linear increase in fragment movements with increasing load (Figure 6). In LCPcaudal, where the plate is located below the spina scapulae, the plate flexed more significantly (*p* < 0.001), resulting in greater fragment movement compared to the other two groups.

### 3.4. Fracture Patterns and Failure Modes

**Figure 7:**
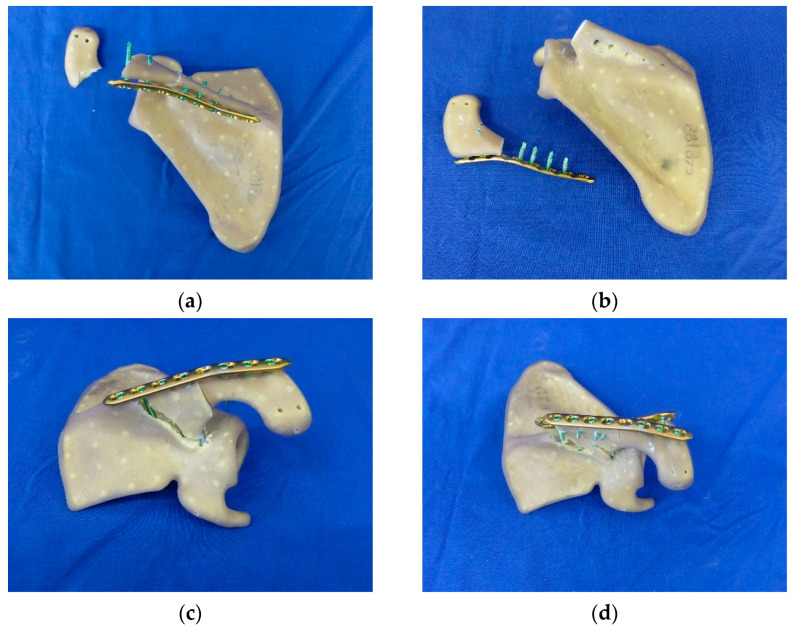
Varying fracture patterns were detected among groups: (**a**) acromion fracture in LCPcaudal, (**b**) failure of medial screws in LCP caudal, (**c**) fracture of spina (LCPdorsal), and (**d**) wedge-shaped fracture from the most medial screw extending to the scapular notch (LCPdouble).

In the LCPcaudal, two different failure modes were detected. In four specimens, the acromion fractured where the two most lateral screws protruded into it (Figure 7a). In one of the two samples, a breakage of the screw could also be detected. The thread of the screw head broke off in the LCP. In two LCPcaudal specimens, the medial screws, which were fixed in the spina scapulae, loosened out during the cyclic loading. The cyclic loading of the trial caused the screws to nick the synthetic bone, resulting in failure of the screw–bone contact. The medial portions of the plate fixation were completely pulled out of the spina scapulae, while the connection in the acromial portion remained intact (Figure 7b).

In five of the six specimens of LCPdorsal, a total breakout of the screws from the spina scapulae including fragmentation of the spina occurred. A wedge-shaped fragment formed between the two screws medial to the fracture gap to be bridged (Figure 7c). In one specimen, a longitudinal fracture of the scapula occurred; however, this specimen fractured at approximately the same position of the screws in the other trials in this group.

Due to the increased number of screws protruding into the spina scapulae from two directions, a stripping of the screws occurred in specimens of LCPdouble, creating a fracture starting from the most medial screw of the plates and extending to the scapular notch. Failure of the screws in the acromial fragment could not be detected (Figure 7d).

## 4. Discussion

The biomechanical performance of three different plating methods of type III acromial fractures were evaluated in terms of stiffness, failure loads, failure modes, as well as interfragmentary motion. Double plating on the caudal and the dorsal aspect of the spina scapulae performed best in all parameters studied, while placing the plate caudally below the spina demonstrated the worst biomechanical performance. If only a single plate was used for fracture fixation, the dorsal placement produced a stiffer fixation compared to the caudal placement without showing an improved strength or survival.

Currently, there is no uniform recommendation in the literature for the treatment of Levy type III acromion fractures. In a biomechanical study, Kicinski et al. investigated the mechanical properties of three different types of plates, all of which were fixed dorsally to the spina scapula. In their test setting, the locking compression plate performed better than a lateral clavicular plate and a reconstruction plate in terms of failure load and maximal displacement at fracture sites. However, the load was applied vertically from the cranial direction and with pressure on the acromion instead of a physiologic muscle pull [15] and also the displacement at the fracture site was evaluated in the caudal direction. Compared to the load protocol of Kicinski et al., longer load cycles and smaller steps of load increase were used in this present study allowing for a more accurate resolution of the failure load.

In a study by Katthagen and colleagues, a similar LCP double plating approach was already investigated; however, their study differs from the present one in the type and dimension of plates used and the type and direction of the load (elevation instead of abduction of the arm). In their study, the double plating approach performed better than the single-plating approach in terms of failure load [15,18,20], which is consistent with the results of this study. From the combined view of Katthagen et al. [15,18,20] and our study, we conclude that double plating is preferable from a biomechanical point of view in terms of failure load, stiffness, and fragment movements compared to a single-plating approach.

Compared to other studies in the field, in our study we placed more emphasis on the correct physiological positioning of the scapula in the biomechanical test setup. By using templates and artificial bones, highly reproducible tests could be performed. Thus, the results of our study are more meaningful than those of other studies. Other studies mainly focused on testing the elevation of the arm [15,18,20], whereas our study is the first to test plate–bone constructs in a 40° abduction setting, enriching the biomechanical perspective of this fracture treatment.

Although there is already a study that has investigated a double plate trial [20], our results complement this study, as we investigated a different movement of the arm, a more physiological positioning of the scapula, and also a cyclic movement to failure of the constructs. In addition, our study provides information about fragment movements in the direction of the muscle pull of the deltoid muscle. Furthermore, our study investigated the comparison of the double-plate approach and additionally two single-plate constructs; this was not the case in other studies [15,18,20].

Double plating showed higher failure loads, lower fragment movements, and higher stiffness in our test compared to the other two groups investigated. This seems to be obvious due to the doubled number of plates and doubled number of screws protruding into the scapula from two different directions which created a very stable construct. In our experiment, the caudal plate showed the highest fragment movements, also because the pulling direction of the muscle acts “obliquely downwards”, thus acting on the narrow side of the plate. The caudally applied plate therefore bends more easily than the dorsally applied plate on the spina scapulae, as in the latter the force of the muscle acts on the wide side of the plate. This seems obvious as the plates are also manually precontored intraoperatively to fit the shape of the spina scapula.

In the LCPdouble group, two plates were used and, therefore, twice the amount of screws were used for fixation. The fractures produced in the trial showed a wedge-shaped fracture, starting from the most medial screw, which runs to the notch of the scapula. This fracture shape is due to the thin and prominent shape of the spina and acromion, but also to the notching effect of the brittle plastics of the Sawbones scapulae.

Together with the double-plating results from the study by Katthagen et al., in which plate treatment of the acromion with elevation of the arm, double plating was investigated and found to be superior to a single plate [20]. Thus, the double plating approach for the treatment of Levy III acromion fractures can be recommended from a biomechanical point of view.

Results after failed open reduction internal fixation (ORIF) or nonunion are very poor, and conservative treatment alone leads to an unsatisfactory outcome after type III acromial fractures after reverse total shoulder arthroplasty [2,13,14]. In this study, double plate osteosynthesis has shown significant biomechanical advantages over single-plate osteosynthesis and potentially decreases failure rates after ORIF. However, double-plate osteosynthesis requires a more extensile approach and detachment of the deltoid muscle, which can potentially lead to muscle weakness. Nevertheless, we think that the more extended detachment of the deltoid is justified by the potentially better chances of bone healing, since bony healing is the prerequisite for an acceptable outcome. One can minimize the extent of muscle detachment by applying the cranial plate first and stabilizing the fracture. The dorsal plate can then be placed over the muscle and fixed without severely detaching the deltoid muscle. In the future, it would be desirable to have a plate system specifically adapted to the anatomy of the spina scapula, which takes the deltoid muscle more into consideration and protects it.

Finally, limitations of this study should also be mentioned. As in most biomechanical studies, due to the absence of the limb, muscles, and other soft tissues, load application had to be abstracted [21] by the pull of the deltoid muscle realized through a testing machine. However, by using a template to place the scapula in the machine, special attention was paid to the physiological (and also reproducible) positioning of the scapula in the testing machine and also to the direction of muscle pull. In this biomechanical test setup, only one muscle traction was considered, that of the pars acromialis of the deltoid muscle, which is mainly responsible for the abduction of the arm. In the test setup, however, the muscle pull during a 40° abduction of the arm was to be simulated. The other two parts of the deltoid muscle, pars acromialis and pars spinalis, are only innervated at an abduction of more than 60° [22]. Therefore, their neglection is well justified.

Another strength of this study was the use of synthetic bone surrogates. These show hardly any geometric and only low mechanical variability [23,24]. This enabled the uniform and reproducible placement of the plates on the specimens as well as the uniform fracturing with the oscillating saw, which was also accomplished with the help of a template. The use of templates for positioning the scapula in the testing machine, cutting the fracture, and positioning the osteosyntheses on the scapula, as well as the use of Sawbones artificial scapulae, allowed highly reproducible tests and comparability between groups. When using human scapulae, the size, gender, and bone density of the donors would influence the mechanical properties of the bones and thus increase the variability of the results [23].

## 5. Conclusions

In conclusion, the double plating approach with two locking plate constructs performed best in all biomechanical parameters for the fixation of type III acromion fractures under simulated arm abduction.

## Data Availability

Data will be provided by the corresponding author upon request.

## References

[1] Zumstein M.A., Pinedo M., Old J., Boileau P. (2011). Problems, complications, reoperations, and revisions in reverse total shoulder arthroplasty: A systematic review. J. Shoulder Elb. Surg..

[2] Hattrup S.J. (2010). The influence of postoperative acromial and scapular spine fractures on the results of reverse shoulder arthroplasty. Orthopedics.

[3] Teusink M.J., Otto R.J., Cottrell B.J., Frankle M.A. (2014). What is the effect of postoperative scapular fracture on outcomes of reverse shoulder arthroplasty?. J. Shoulder Elb. Surg..

[4] Ascione F., Kilian C.M., Laughlin M.S., Bugelli G., Domos P., Neyton L., Godeneche A., Edwards T.B., Walch G. (2018). Increased scapular spine fractures after reverse shoulder arthroplasty with a humeral onlay short stem: An analysis of 485 consecutive cases. J. Shoulder Elb. Surg..

[5] Otto R.J., Virani N.A., Levy J.C., Nigro P.T., Cuff D.J., Frankle M.A. (2013). Scapular fractures after reverse shoulder arthroplasty: Evaluation of risk factors and the reliability of a proposed classification. J. Shoulder Elb. Surg..

[6] King J.J., Dalton S.S., Gulotta L.V., Wright T.W., Schoch B.S. (2019). How common are acromial and scapular spine fractures after reverse shoulder arthroplasty? A systematic review. Bone Jt. J..

[7] Brusalis C.M., Taylor S.A. (2020). Periprosthetic Fractures in Reverse Total Shoulder Arthroplasty: Current Concepts and Advances in Management. Curr. Rev. Musculoskelet. Med..

[8] Levy J.C., Anderson C., Samson A. (2013). Classification of postoperative acromial fractures following reverse shoulder arthroplasty. J. Bone Jt. Surg. Am..

[9] Nyffeler R.W., Altioklar B., Bissig P. (2020). Causes of acromion and scapular spine fractures following reverse shoulder arthroplasty: A retrospective analysis and literature review. Int. Orthop..

[10] Schenk P., Aichmair A., Beeler S., Ernstbrunner L., Meyer D.C., Gerber C. (2020). Acromial Fractures Following Reverse Total Shoulder Arthroplasty: A Cohort Controlled Analysis. Orthopedics.

[11] Mayne I.P., Bell S.N., Wright W., Coghlan J.A. (2016). Acromial and scapular spine fractures after reverse total shoulder arthroplasty. Shoulder Elb..

[12] Crosby L.A., Hamilton A., Twiss T. (2011). Scapula fractures after reverse total shoulder arthroplasty: Classification and treatment. Clin. Orthop. Relat. Res..

[13] Patterson D.C., Chi D., Parsons B.O., Cagle P.J. (2019). Acromial spine fracture after reverse total shoulder arthroplasty: A systematic review. J. Shoulder Elb. Surg..

[14] Neyton L., Erickson J., Ascione F., Bugelli G., Lunini E., Walch G. (2019). Grammont Award 2018: Scapular fractures in reverse shoulder arthroplasty (Grammont style): Prevalence, functional, and radiographic results with minimum 5-year follow-up. J. Shoulder Elb. Surg..

[15] Kicinski M., Puskas G.J., Zdravkovic V., Jost B. (2018). Osteosynthesis of type III acromial fractures with locking compression plate, lateral clavicular plate, and reconstruction plate: A biomechanical analysis of load to failure and strain distribution. J. Shoulder Elb. Surg..

[16] Umehara J., Yagi M., Hirono T., Komamura T., Nishishita S., Ichihashi N. (2019). Relationship between scapular initial position and scapular movement during dynamic motions. PLoS ONE.

[17] Sakoma Y., Sano H., Shinozaki N., Itoigawa Y., Yamamoto N., Ozaki T., Itoi E. (2011). Anatomical and functional segments of the deltoid muscle. J. Anat..

[18] Spiegl U.J., Smith S.D., Todd J.N., Wijdicks C.A., Millett P.J. (2015). Biomechanical evaluation of internal fixation techniques for unstable meso-type os acromiale. J. Shoulder Elb. Surg..

[19] Ackland D.C., Wu W., Thomas R., Patel M., Page R., Sangeux M., Richardson M. (2019). Muscle and Joint Function After Anatomic and Reverse Total Shoulder Arthroplasty Using a Modular Shoulder Prosthesis. J. Orthop. Res..

[20] Katthagen J.C., Sußiek J., Frank A., Wermers J., Schliemann B., Raschke M.J. (2021). Double plating is associated with higher fixation strength than single plating in osteoporotic fractures of the scapular spine: A biomechanical study. Arch. Orthop. Trauma Surg..

[21] Augat P., Hast M.W., Schemitsch G., Heyland M., Trepczynski A., Borgiani E., Russow G., Märdian S., Duda G.N., Hollensteiner M. (2021). Biomechanical models: Key considerations in study design. OTA Int..

[22] Schünke M. (2019). 5 Schultergürtel und Schultergelenk (I). Anatomie für Osteopathen.

[23] Gardner M.J., Silva M.J., Krieg J.C. (2012). Biomechanical testing of fracture fixation constructs: Variability, validity, and clinical applicability. J. Am. Acad. Orthop. Surg..

[24] Hollensteiner M., Sandriesser S., Hackl S., Augat P. (2021). Custom-made polyurethane-based synthetic bones mimic screw cut-through of intramedullary nails in human long bones. J. Mech. Behav. Biomed. Mater..

